# Dynamics of *P. vivax* clones in a cohort of children with or without primaquine treatment at baseline

**DOI:** 10.1186/1475-2875-13-S1-O24

**Published:** 2014-09-22

**Authors:** Rahel Wampfler, Leanne Robinson, Natalie Hofmann, Andreea Waltmann, Inoni Betuela, Mariabeth Silkey, Peter Siba, Tom Smith, Ivo Mueller, Ingrid Felger

**Affiliations:** 1 Swiss Tropical and Public Health Institute, Basel, Switzerland; 2 University of Basel, Basel, Switzerland; 3 Walter and Eliza Hall Institute, Parkville, Australia; 4 PNG Institute of Medical Research, Madang & Maprik, Papua New Guinea; 5 Department of Medical Biology, University of Melbourne, Victoria, Australia; 6 Centre de Recerca en Salut Internacional de Barcelona (CRESIB), Barcelona, Spain

## 

*P. vivax* was detected by PCR in 45% of children aged 5-10 years from our study area in Papua New Guinea (PNG). 504 children were randomized into 2 arms according to Primaquine (PQ) treatment or not at baseline and actively and passively followed for 9 months. We genotyped all *P. vivax* infections, the majority of these being multi-clone infections. All blood samples positive for *P. vivax* by qPCR were tested for gametocyte carriage by targeting *pvs25* transcripts. Primaquine reduced the risk of *P. vivax* infections by 80%. The multiplicity of infection and the density of asexual *P. vivax* stages were not significantly different in both treatment arms. The number of new clones (force of blood-stage infection) was 2.38 ± 0.17 per person per year-at-risk in the PQ-arm compared to 8.04 ± 0.41 in the Placebo arm (*P* < 0.05). The duration of infections did not differ between the treatment arms, with 73 days [95% CI: 33-849] and 68 days [95% CI: 40-247] in the PQ or Placebo arm, respectively. Detectability of *P. vivax* clones was low with 0.26 ± 0.06 and 0.24 ± 0.04 in the PQ and Placebo arms. PQ-treated children had a 75% lower risk of carrying gametocytes compared to Placebo recipients. *P. vivax* positive children in both arms were equally likely to show gametocyte positivity. We conclude that *P. vivax* relapses contribute significantly to the high burden of *P. vivax* infection and transmission in PNG. All other infection dynamics parameters were consistent between treatment arms and apparent relapses behave like new infections.

